# Olfactory dysfunction as an early pathogenic indicator in *C. elegans* models of Alzheimer's and polyglutamine diseases

**DOI:** 10.3389/fnagi.2024.1462238

**Published:** 2024-10-01

**Authors:** Weikang Xue, Ziyi Lei, Bin Liu, Hanxin Guo, Weiyi Yan, Youngnam N. Jin, Yanxun V. Yu

**Affiliations:** ^1^Department of Neurology, Medical Research Institute, Zhongnan Hospital of Wuhan University, Wuhan University, Wuhan, China; ^2^Frontier Science Center for Immunology and Metabolism, Wuhan University, Wuhan, China

**Keywords:** neurodegenerative diseases, Alzheimer's disease, polyglutamine diseases, *C. elegans*, neurons, olfaction

## Abstract

Neurodegenerative diseases such as Alzheimer's disease and polyglutamine diseases are characterized by abnormal accumulation of misfolded proteins, leading to neuronal dysfunction and subsequent neuron death. However, there is a lack of studies that integrate molecular, morphological, and functional analyses in neurodegenerative models to fully characterize these time-dependent processes. In this study, we used *C. elegans* models expressing Aβ1-42 and polyglutamine to investigate early neuronal pathogenic features in olfactory neurons. Both models demonstrated significant reductions in odor sensitivity in AWB and AWC chemosensory neurons as early as day 1 of adulthood, while AWA chemosensory neurons showed no such decline, suggesting cell-type-specific early neuronal dysfunction. At the molecular level, Aβ1-42 or Q40 expression caused age-dependent protein aggregation and morphological changes in neurons. By day 6, both models displayed prominent protein aggregates in neuronal cell bodies and neurites. Notably, AWB neurons in both models showed significantly shortened cilia and increased instances of enlarged cilia as early as day 1 of adulthood. Furthermore, AWC neurons expressing Aβ1-42 displayed calcium signaling defects, with significantly reduced responses to odor stimuli on day 1, further supporting early behavioral dysfunction. In contrast, AWA neuron did not exhibit reduced calcium responses, consistent with the absence of detectable decreases in olfactory sensitivity in these neurons. These findings suggest that decreased calcium signaling and dysfunction in specific sensory neuron subtypes are early indicators of neurodegeneration in *C. elegans*, occurring prior to the formation of visible protein aggregates. We found that the ER unfolded protein response (UPR) is significantly activated in worms expressing Aβ1-42. Activation of the AMPK pathway alleviates olfactory defects and reduces fibrillar Aβ in these worms. This study underscores the use of *C. elegans* olfactory neurons as a model to elucidate mechanisms of proteostasis in neurodegenerative diseases and highlights the importance of integrated approaches.

## 1 Introduction

The extended human lifespan has led to a rise in age-related neurodegenerative diseases, including Alzheimer's disease (AD), frontotemporal dementia (FTD), Parkinson's disease (PD), Huntington's disease (HD), and amyotrophic lateral sclerosis (ALS) (Wilson et al., 2023). The growing number of patients places a significant burden on families and rising societal costs.

AD is a chronic, progressive neurological disorder and the most prevalent form of dementia, with its incidence rising with age. AD affects various brain regions, such as hippocampus and cortex, and is characterized by senile plaques primarily composed of the amyloid β-protein (Aβ) and neurofibrillary tangles made by hyperphosphorylated tau protein, resulting in memory loss and cognitive impairment (Breijyeh and Karaman, 2020; Knopman et al., 2021). Less than 5% of AD cases are familial. Familial AD is caused by mutations in amyloid precursor protein (APP) and presenilin 1/2 (PSEN-1/2) genes, while the apolipoprotein E (APOE) gene is the most significant genetic risk factor for sporadic AD. The cause of AD is a complex interplay of genetic and environmental factors (Breijyeh and Karaman, 2020; Knopman et al., 2021). Major pathogenic hypotheses include Aβ deposit, tau propagation, impaired cholinergic neurons, inflammation, and oxidative stress. Aβ forms toxic oligomers, fibrils, and plaques, leading to increased ROS, impaired cellular functions, and cell death (Liu et al., 2019). There is no cure, but current treatments focus on managing symptoms and slowing progression.

Polyglutamine (polyQ) diseases encompass a group of neurodegenerative disorders caused by expanded CAG repeats in genes, leading to elongated polyQ tracts within proteins. HD, among the most studied polyQ disorders, results from abnormal expansion of CAG repeats in the huntingtin (HTT) gene (Fan et al., 2014), typically longer than 36 in affected individuals (Bates et al., 2015; Tabrizi et al., 2020). The expanded polyQ tract disrupts protein function, causing protein misfolding and aggregation in the cytosol and nucleus, leading to disrupted proteostasis and ribotoxicity (Gidalevitz et al., 2006; Aviner et al., 2024). Despite HTT being widely expressed in most tissues, this “toxic gain of function” selectively targets medium spiny neurons in the striatum, a brain region crucial for movement control. As the disease progresses, the striatum shrinks, and neurons die. HD symptoms include progressive loss of motor control, cognitive decline, and psychiatric issues. HD exemplifies how CAG repeat expansions trigger neurodegeneration (Lieberman et al., 2019; Malik et al., 2021). Other polyQ diseases, such as spinal and bulbar muscular atrophy (SBMA) and spinocerebellar ataxias (SCAs), share a common feature of an abnormal polyQ tract leading to neuronal dysfunction and/or death.

*C. elegans*, a simple worm, offers a powerful and cost-effective platform for studying human diseases, particularly neurodegenerative diseases (Shen et al., 2018; Roussos et al., 2023; Wu et al., 2024; Yamamoto et al., 2024). Its short lifespan, transparent body, and well-mapped nervous system make it ideal for observing disease processes at both the cellular and whole-animal levels, as well as for precise neuronal tracking and analyses. *C. elegans* shares evolutionarily conserved key molecular pathways with mammals, including humans, enabling researchers to simulate disease models and dissect disease mechanisms. Additionally, its ease of genetic manipulation allows for high-throughput drug and genetic screens, accelerating the discovery of potential treatments. Overall, *C. elegans* serves as a versatile tool for dissecting neurodegenerative diseases at the molecular level, paving the way for new therapeutic strategies.

The first AD model in *C. elegans* was created by expressing human Aβ1–42 in muscle tissue (Link, 1995). While this approach did not directly mimic human AD, it provided valuable insights. The worms rapidly formed Aβ deposits, exhibited progressive paralysis, and died within 2–3 days, demonstrating Aβ's ability to form toxic structures and cause cellular damage, similar to what is observed in human disease. However, this model more closely resembled inclusion body myositis (IBM) due to its expression in muscle tissue. To better replicate the age-related behavioral dysfunctions seen in human AD, a novel strain with constitutive pan-neuronal Aβ1–42 expression (GRU102) was developed to study the early events of Aβ-mediated toxicity, with a focus on mitochondrial metabolism. GRU102, like previous models, exhibits mild phenotypes such as chemotaxis failure and age-related neuromuscular defects. Despite Aβ aggregates appearing only in older worms (day 12), young GRU102 already showed defects in energy metabolism and reduced mitochondrial energetics (Fong et al., 2016; Teo et al., 2019).

PolyQ expansions disrupt protein quality control, disturbing the delicate balance within cells and impairing the folding and proper function of many proteins (Gidalevitz et al., 2006; Aviner et al., 2024). This underscores the widespread consequences of polyQ toxicity. *C. elegans* worms offer a powerful tool to study these effects and disease mechanisms. Transgenic *C. elegans* models have been developed to express polyQ of varying lengths in different neuron types, recapitulating features of diseases like HD. The length of the polyQ repeat directly influences disease onset and severity. Notably, some models show cellular dysfunction mediated by mutant polyQ proteins even before protein aggregation occurs, suggesting that these early disruptions may be crucial in disease progression (Faber et al., 1999). For example, expressing mutant Htt in touch receptor neurons using the mec-3 promoter revealed perinuclear aggregates and axonal abnormalities without cell death (Parker et al., 2001). Pan-neuronal expression of polyQ under the rgef-1 promoter demonstrated that polyQ repeat size correlates with neuronal dysfunction, requiring more than 40 glutamines for insoluble aggregate formation (Brignull et al., 2006). Muscle-specific models expressing polyQ in body wall muscle cells showed reduced motility and lifespan, with aggregation and toxicity increasing with age. Consistently, a length of 35–40 glutamines was identified as critical for aggregation and dysfunction (Morley et al., 2002; Lee et al., 2017). These *C. elegans* models offer valuable insights into the broad impact of polyQ expansion and mechanisms underlying disease progression.

Olfactory dysfunction is a common and early manifestation in many neurodegenerative diseases, including AD, HD, and SCAs (Abele et al., 2003; Murphy, 2019; Laroche et al., 2020; Pacyna et al., 2023; Hawkes, 2003). Understanding how neurodegeneration disrupts sensory perception can offer crucial insights into disease progression and potential therapeutic strategies. In this study, we utilize two *C. elegans* neuronal models for AD (Fong et al., 2016) and polyglutamine diseases (Brignull et al., 2006) to investigate early olfactory pathogenic events and delineate disease phenotypes across different age stages, reflecting the progressive nature of these neuronal diseases. We also investigated which cellular compartments are most susceptible to pathological protein aggregation and examined the involvement of the AMPK pathway in *C. elegans* models of neurodegeneration.

## 2 Materials and methods

### 2.1 *C. elegans* and drug treatment

All *C. elegans* strains were cultured on nematode growth medium (NGM) plates seeded with Escherichia coli strain OP50 at 20°C according to established protocols (Brenner, 1974). For drug treatment, AICAR (Targetmol, CAS 2627-69-2) or metformin (Sangon Biotech, CAS 1115-70-41) was dissolved in Milli-Q water and added to NGM agar to achieve final concentrations of 1 μM or 10 μM (AICAR) or 2 mM or 25 mM (metformin) to pour plates. Twenty-five young adult *C. elegans* were placed on these plates to lay eggs for 4 h. After removing the worms, the plates were incubated at 20°C for 4 days, allowing the eggs to develop into day 1 adults for subsequent analysis.

### 2.2 Generation of transgenic worms

Transgenic strains were generated using standard genetic procedures (Mello et al., 1991), with plasmids injected at concentrations of 20–50 ng/μl. Well-fed, day 1 young adult worms were utilized for this procedure. These worms were placed on a dried agarose pad to immobilize them. A drop of halocarbon oil was quickly added to cover the worms from drying out. Using a microinjection setup, a mixed plasmid DNA solution was carefully injected into the gonads of the worms. Following injection, the worms were allowed to recover on NGM plates at 20°C. Transgenic animals were identified based on expression of a fluorescent co-injection marker or other selectable traits. These transgenic worms were then selected and transferred to fresh plates to establish lines, ensuring the gene of interest was transmitted to subsequent generations. At least three independent transgenic lines were maintained for further experimentation. A comprehensive list of strains is provided in Supplementary Table 1.

### 2.3 Chemotaxis assays

Chemotaxis assays were performed following a previously described protocol (Bargmann et al., 1993) with slight modifications. The assay plates contained 1.6% agar, 25 mM potassium phosphate (pH 6.0), 1 mM calcium chloride, and 1 mM magnesium sulfate. The solution was microwaved until the agar completely melted and was then allowed to cool to ~60°C. Subsequently, 10 ml of the solution was poured into each 9 cm Petri plate, and the plates were left uncovered to dry for 1 h at room temperature. At the onset of the assay, ~50 synchronized young adult worms were picked and placed onto the center of the freshly prepared assay plate. Two 1 μl spots of odorant and diluent ethanol were placed at opposite ends of the plate, along with 1 μl of 1 M potassium azide at each spot to immobilize the worms. The odorants used were as follows: isoamyl alcohol (Cat#M823039, Macklin), 2-butanone (Cat#80022818, Sinopharm Chemical Reagent), diacetyl (Cat#B85307, Sigma-Aldrich), pyrazine (Cat#P109613, Aladdin Scientific Corp), and 2-nonanone (Cat#N814618, Macklin), all diluted in ethanol. After 60 min, the worms anesthetized by potassium azide were counted. The chemotaxis index was calculated as: (number of animals at the odor spot—number of animals at the counter spot)/total number of animals on the plate. Chemotaxis indexes are presented as mean ± s.e.m., with individual data points shown in all figures.

### 2.4 Western blot

Age-synchronized worms were collected and washed with M9 buffer. The worms were homogenized in lysis buffer containing protease inhibitors. The homogenate was centrifuged at 15,000 rpm for 15 min at 4°C. The supernatant was collected, and protein concentration was measured using BCA assay. Samples were normalized to equal protein concentration, mixed with loading buffer, and boiled for 10 min at 100°C. Western blotting was performed according to standard protocols. Equal amounts of protein lysate (10 or 20 μg) were loaded onto a 12% SDS-PAGE gel and separated in electrophoresis. Proteins were then transferred to a PVDF membrane, blocked with 5% non-fat milk, and incubated overnight at 4°C with primary antibodies. The membrane was washed and incubated with HRP-conjugated secondary antibodies for 1 h at room temperature. After additional washes, the membrane was incubated with ECL solution and exposed to film. The primary antibodies used were: anti-GFP Rabbit pAb (ABclonal, AE011, 1:20,000) and anti-β-Actin Mouse mAb (ABclonal, AC004, 1:5,000).

### 2.5 Dot blot

Dot blot assays were performed following a similar procedure to western blotting, with the exception that 2 μl of sample containing 4 μg of protein were loaded directly onto the nitrocellulose membrane without electrophoresis and allowed to dry at room temperature for 30 min. Blots were stained with Ponceau red as a total protein level indicator. The membrane was then blocked with 5% non-fat milk, incubated overnight at 4°C with primary antibodies, and processed as in western blotting. The primary antibodies used were: anti-β-Amyloid oligomers [6E10] antibody (BioLegend, 803004, 1:20000) and anti-Amyloid fibrils [mOC22] antibody (Abcam, ab205339, 1:20000).

### 2.6 Fluorescence imaging

Freshly prepared 1% agarose pads were made on glass slides. Fluorescent transgenic worms cultured on NGM plates were collected for imaging. The worms were anesthetized with 10 μM levamisole, arranged on the agarose pads, and covered with a cover slip. Imaging was performed using a Nikon Ti2-E inverted microscope with a 100X oil objective to observe polyQ aggregation and cilia morphology. Z-stack images were captured with 0.5 μm step size. The maximum projection image of each worm was examined visually for exceptionally bright puncta of subcellular size, which were defined as aggregates. The total number of aggregates was then determined by counting all aggregation puncta in the maximum projection images. Neurites were categorized as having “no aggregation” if all neuronal processes appeared smooth and free of puncta.

### 2.7 Calcium imaging

Calcium imaging experiments were performed as described in Chalasani et al., with some modifications. Transgenic worms expressing the calcium sensor GCaMP6s in specific neuron were loaded into a custom-made microfluidic chip. The animal's nose was exposed to a stream of liquid that could be manually switched between diluted odor and buffer. The buffer consisted of 25 mM potassium phosphate (pH 6.0), 1 mM CaCl_2_, and 1 mM MgSO_4_. Odorants were freshly diluted in the buffer. Before recordings, the nose tips of worms trapped in the chip were allowed to adapt for 2 min in the buffer solution stream. One channel containing 2 nM fluorescein was used to confirm correct fluid flow. Imaging was conducted on a ZEISS inverted microscope with a 40X water objective and an sCMOS camera (PCO. Edge 4.2 bi). Time-lapse images were recorded at 1 frame per second.

### 2.8 Real-time qPCR

Approximately 100 day 1 worms were washed three times with M9 buffer and collected in RNAiso Plus (Takara). Total RNA was extracted following the manufacturer's protocol. cDNA libraries were synthesized using HiScript III RT SuperMix with gDNA wiper (Vazyme). Subsequently, qPCR amplification was carried out on a CFX Connect Real-Time PCR system (Bio-Rad) using 2x Taq Pro Universal SYBR qPCR Master Mix (Vazyme). The gene expression levels were quantified by the ΔΔCt method and normalized against actin (*act-1*). Each sample was assayed in triplicate.

### 2.9 Quantification and statistical analysis

Dot blot and western blot signal intensities were quantified using ImageJ software. The Neuroanatomy plugin in Fiji software was used to measure cilia length by identifying both ends of the cilia and calculating their length. Cilia enlargement was defined as a cilia width of cilia visually greater than the average width observed in wild-type animals. Calcium image processing was performed using custom scripts on MATLAB (Mathworks). Calcium image intensity was processed by custom script “calcium imaging analysis”, and the heatmaps for calcium imaging analysis were created by custom script “HotMap Create”. The fluorescence intensity during the first 15 s time window before the odorant stimulus was averaged and defined as F0. Fluorescence intensity change ΔF/F0 was calculated as (background-corrected fluorescence – F0)/F0. Calcium responses are presented as average changes, with shaded regions indicating s.e.m., and heatmaps of individual traces. All statistical analyses were performed using GraphPad Prism 8. Differences between two groups were assessed using unpaired *t*-tests. Multigroup data was analyzed by one-way ANOVA with Tukey's multiple comparisons test or two-way ANOVA with Sidak's multiple comparisons test. *P* < 0.05 was considered statistically significant. ^*^P < 0.05; ^**^P < 0.01; ^***^P < 0.001. ^****^P < 0.0001; n.s., not significant.

## 3 Results

### 3.1 Olfactory dysfunction in *C. elegans* models of neurodegenerative diseases

*C. elegans* has three pairs of primary olfactory neurons: AWA, AWB, and AWC (Bargmann, 2006; Ferkey et al., 2021). Among them, AWA and AWC neurons mediate attraction to attractive odors, while AWB neurons mediate avoidance of repulsive odors (Troemel et al., 1997; Bargmann, 2006). We investigated the impact of expressing neurodegenerative proteins on olfactory behavior using an array of odors primarily detected by these three pairs of olfactory neurons.

**Figure 1:**
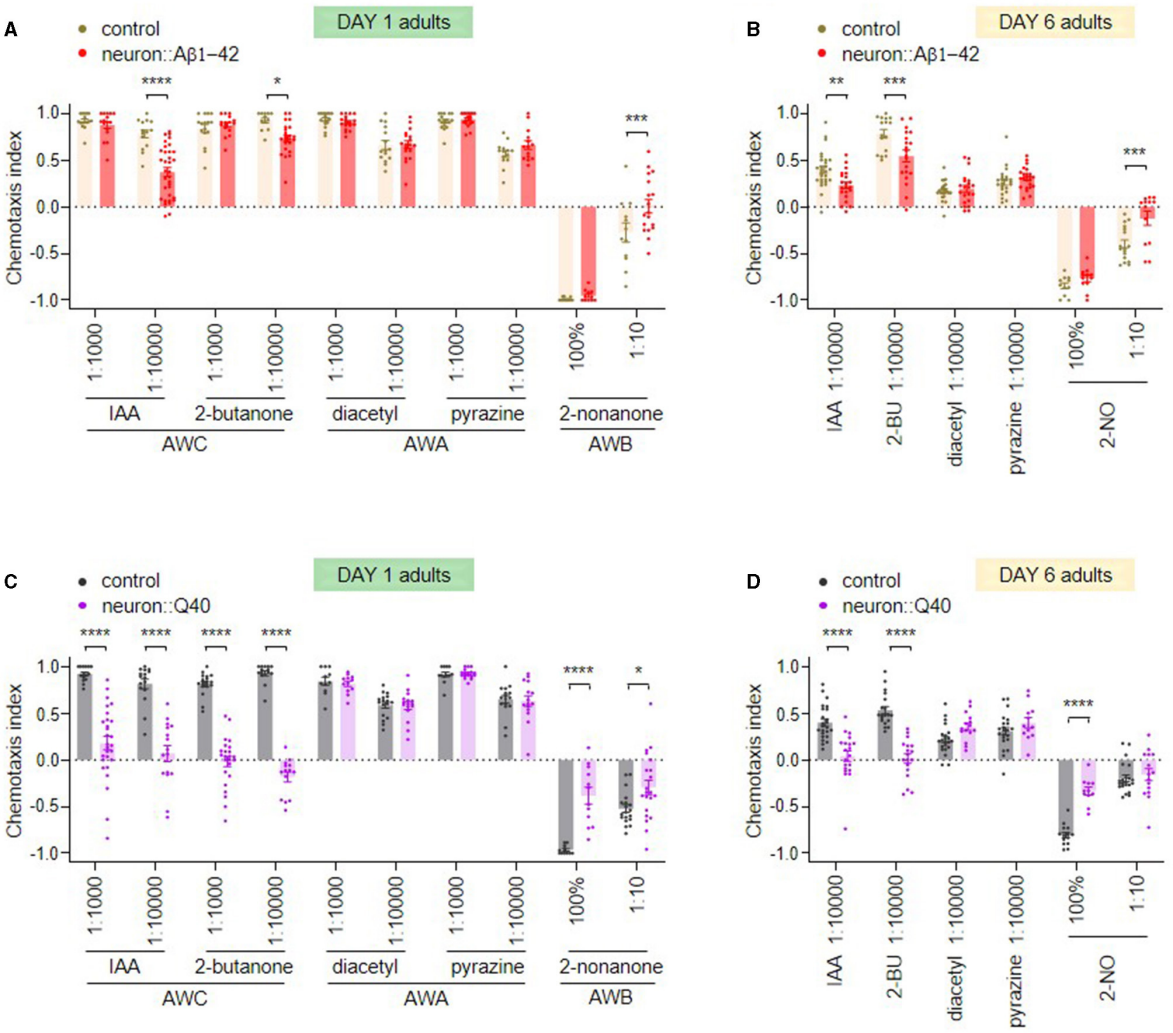
Olfactory dysfunction in *C. elegans* models of neurodegenerative diseases. **(A**, **B)** Chemotaxis responses of wild-type adults and adults with pan-neuronal expression of Aβ1-42 to various odors on the first **(A)** and sixth **(B)** days of adulthood. **(C**, **D)** Chemotaxis responses of wild-type adults and adults with pan-neuronal expression of Q40 to various odors on the first **(C)** and sixth **(D)** days of adulthood. Data are from three or more independent experiments, and are presented as mean ± s.e.m. *P* values are derived from two-way ANOVA with Sidak's multiple comparisons test. ^*^P < 0.05; ^**^P < 0.01; ^***^P < 0.001; ^****^P < 0.0001; n.s., not significant. 2-BU is 2-butanone, and 2-NO is 2-nonanone.

Compared to control animals, day 1 adult *C. elegans* expressing Aβ1-42 in all neurons, an AD model used in this study, exhibited slightly reduced olfactory sensitivity to odors detected by AWC and AWB neurons, including isoamyl alcohol (IAA), 2-butanone and 2-nonanone, but showed no difference from the control in response to odors sensed by AWA neurons including diacetyl and pyrazine (Figure 1A). To assess age-dependent decline in olfactory function, we tested day 6 adults, which have largely ceased reproduction. We found that control day 6 adults already exhibited decreased olfactory sensitivity across all tested odors. Interestingly, day 6 adults expressing Aβ1-42 displayed significantly greater defects in AWC and AWB odor responses compared to age-matched controls, while their sensitivity to AWA odors remained unaffected (Figure 1B).

*C. elegans* expressing 40 polyglutamine repeats (Q40) fused with YFP in all neurons, a polyQ model used in this study, displayed significant olfactory behavior defects in detecting odors sensed by AWC and AWB neurons. Interestingly, responses to AWA-sensed odors remained intact. Furthermore, the Q40 model exhibited more severe olfactory defects than the AD model. While both models showed defects in AWC and AWB odor responses, Q40 worms displayed a near-complete loss of chemotaxis toward AWC-sensed odors even in day 1 adults (Figure 1C). While wild-type day 6 adults exhibited a decline in olfactory sensitivity compared to younger stages, day 6 adults expressing Q40 displayed significantly greater olfactory dysfunction in AWC and AWB responses compared to age-matched controls (Figure 1D). Notably, similar to the AD model, Q40 worms maintained functional AWA olfaction at both day 1 and day 6, suggesting a differential susceptibility of olfactory neurons to protein aggregation.

### 3.2 Age-dependent protein aggregation in *C. elegans* models of neurodegeneration

A hallmark feature of neurodegenerative diseases is the abnormal accumulation of misfolded proteins, which aggregate and form insoluble fibrils as the disease progresses. These aggregates disrupt normal cellular functions, leading to neuronal dysfunction and eventual cell death. The presence of these protein aggregates is a characteristic pathological feature across various neurodegenerative diseases (Wilson et al., 2023).

**Figure 2:**
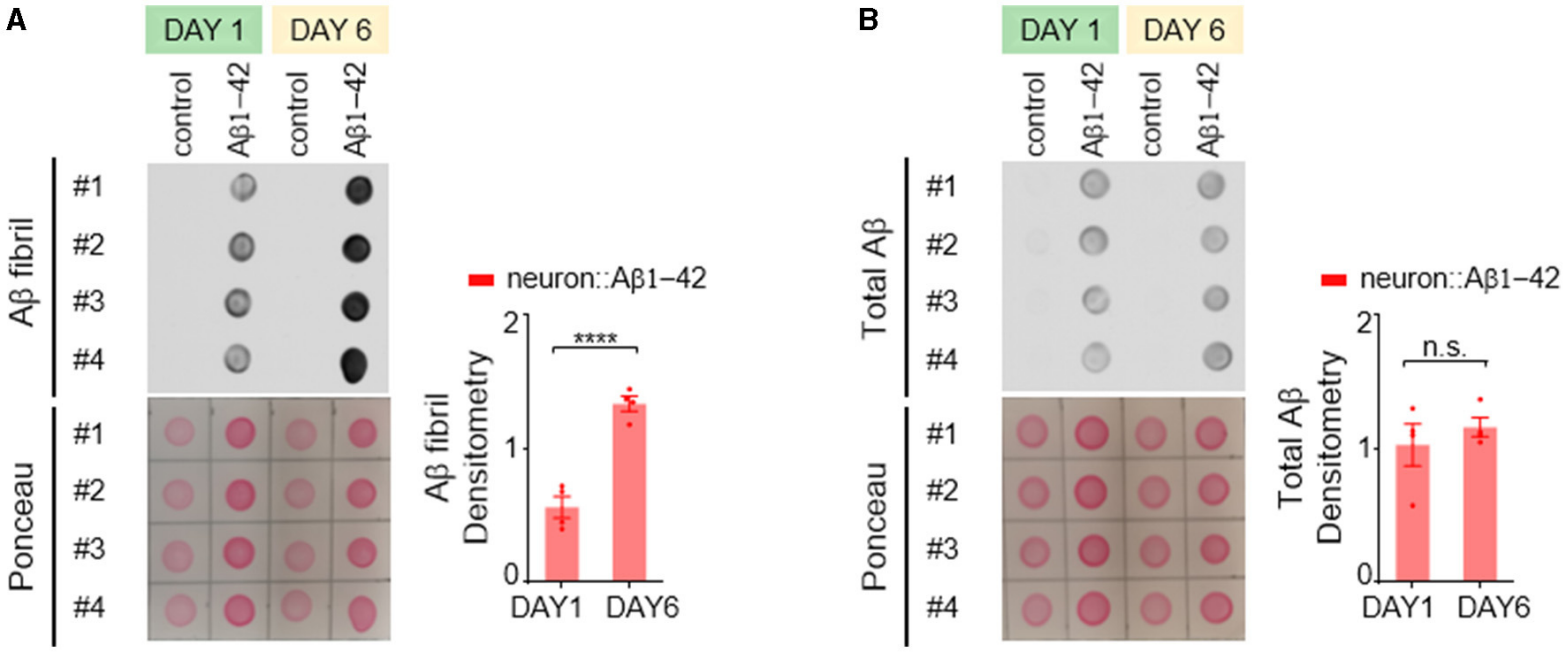
Aβ1-42 forms fibrillar aggregates in *C. elegans* neurons. **(A**, **B)** Fibril Aβ levels **(A)** and total Aβ levels **(B)** in worms were measured using a dot blot assay on the first or sixth day of adulthood. Total Aβ levels remained constant from day 1 to day 6, but fibril Aβ levels increased significantly. Aβ densitometry was quantified on the right. Data are presented as mean ± s.e.m. *P* values are derived from unpaired *t*-test. ****P < 0.0001; n.s., not significant.

In the *C. elegans* model expressing Aβ1-42, we employed an antibody specific for the fibrillar form of Aβ protein to investigate its aggregation patterns. We observed a significant age-dependent increase in fibrillar Aβ aggregates within day 6 adult worms compared to their day 1 counterparts (Figure 2A). Interestingly, the total amount of Aβ protein remained relatively constant across these two age groups (Figure 2B), indicating that the progressive accumulation of fibrillar Aβ is not due to an increased expression level.

**Figure 3:**
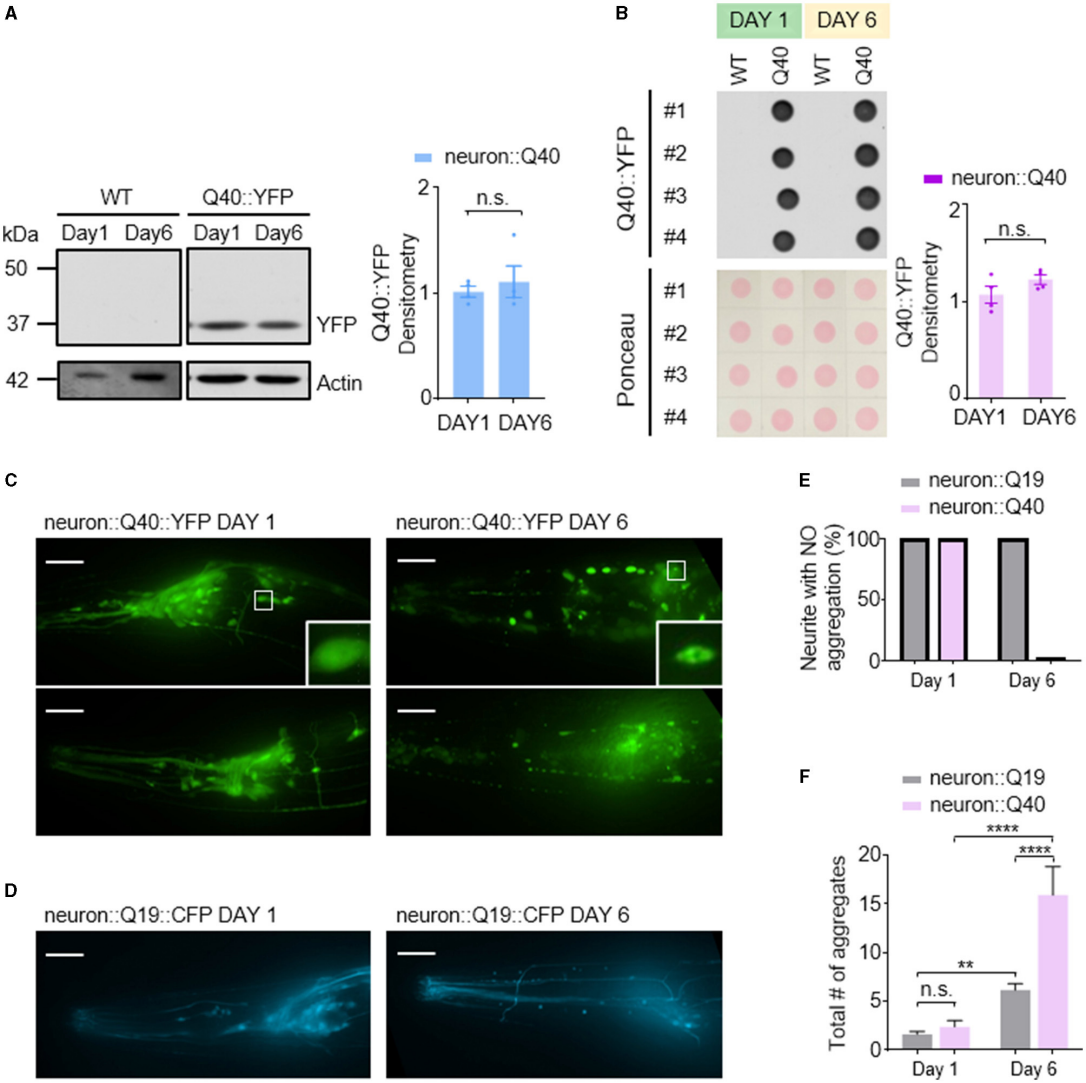
Q40 forms soluble aggregates in the neuronal cell bodies and processes of *C. elegans*. **(A**, **B)** Q40::YFP levels were measured using either western blot **(A)** or dot blot **(B)** assays on the first or sixth day of adulthood. Quantification of Q40::YFP densitometry is shown on the right. No higher molecular weight bands than the monomer protein band (~32kD) were observed in the western blot. **(C)** By day 6, *C. elegans* expressing 40 polyglutamine (Q40) showed a significant increase in polyQ aggregates in neuronal cell bodies or processes in head neurons. **(D)** In contrast, *C. elegans* expressing 19 polyglutamine (Q19) exhibited only a few aggregates in neuronal cell bodies and none in neurites. **(E**, **F)** The percentage of neurites with no aggregation **(E)** and the total number of aggregates in neuron cell bodies **(F)** were quantified from data obtained from three or more independent experiments. Data are presented as mean ± s.e.m. **(B, F)**. *P* values are derived from unpaired t-test **(B)** and one-way ANOVA with Tukey's multiple comparisons test **(F)**. **P < 0.01; ****P < 0.0001; n.s.: not significant. The scale bar is 20 μm for all confocal images.

In contrast to the Aβ1-42 model, *C. elegans* expressing Q40::YFP did not exhibit any detectable formation of insoluble protein aggregates at either day 1 or day 6 adulthood, as visualized by western blotting (Figure 3A). Western blot analysis showed the absence of higher molecular weight bands compared to the expected size of the monomeric Q40::YFP protein (~32 kDa) (Figure 3A), and the total amount of Q40 did not increase from day 1 to day 6 (Figure 3B).

Despite the lack of detectable insoluble aggregates, *C. elegans* expressing Q40::YFP displayed an age-dependent accumulation of soluble protein aggregates within neurons. Aggregation was defined as the presence of puncta in neurites, while smooth neurites were classified as showing no aggregation. In day 1 worms, YFP fluorescence appeared diffusely distributed throughout most neurons (Figure 3C left). In contrast, day 6 adults exhibited visible Q40::YFP aggregates within neuronal cell bodies and along the neurites of sensory neurons (Figure 3C right). In comparison, the Q19::CFP model, which has a shorter polyglutamine repeat, exhibited minimal aggregation within neuronal cell bodies at day 6 and a near absence of aggregates along neurites (Figures 3D–F). This finding further demonstrates that the length of the polyglutamine repeat influences the severity of protein aggregation.

### 3.3 Ciliary abnormalities in *C. elegans* models of neurodegenerative diseases

Sensory dysfunction can arise from a variety of underlying mechanisms. In sensory neurons, nonmotile primary cilia, hair-like structures protruding from the dendrite tips, play a crucial role in detecting environmental stimuli. To investigate whether defects in sensory behavior observed in our neurodegenerative models stem from ciliary dysfunction, we investigated the morphology of cilia in the AWB sensory neurons. These neurons possess a characteristic fork-shaped cilium, facilitating quantification of its length and observation of potential enlargements (Perkins et al., 1986).

**Figure 4:**
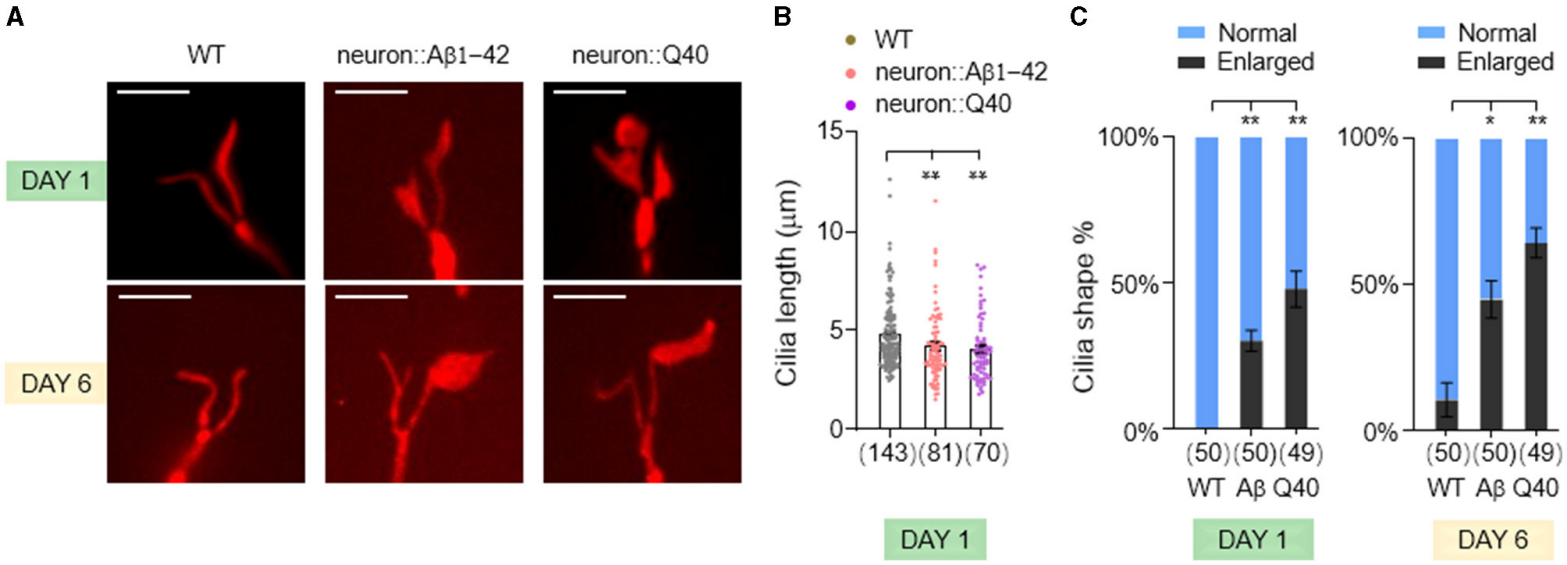
The morphology of AWB neuron cilia was altered in *C. elegans* models of neurodegeneration. **(A)** Visualization of the cilia of AWB neurons using *str-1*p::*myr*-*mNeptune2.5* transgene expression in wild-type adults (left panels), adults with pan-neuronal expressing of Aβ1-42 (middle panels), and adults expressing Q40 (right panels) at day 1 and 6. **(B)** Quantification of cilia length in day 1 adults. Data are mean ± s.e.m. **(C)** Percentage of normal and enlarged cilia in day 1 and 6 adults. Data represent results from two or more independent experiments **(B, C)**, and are presented as mean ± s.e.m. **(B, C)**. The number of cilia assayed is indicated in parentheses below each bar **(B, C)**. *P* values are derived from unpaired *t*-test. *P < 0.05; **P < 0.01; n.s., not significant. The scale bar is 5 μm for all confocal images.

We found a slight but statistically significant decrease in AWB cilia length in both Aβ1-42 and Q40 expressing worms (Figures 4A, B). Notably, we also observed increased instances of enlarged cilia in these mutants (Figures 4A, C), which became more pronounced by day 6. This phenomenon could represent a cilia-specific compensatory mechanism, where the cilia undergo adaptive remodeling to increase their surface area and enhance sensory sensitivity in response to diminished sensory signaling in the neurodegenerative models (Mukhopadhyay et al., 2008). These findings suggest that ciliary morphology defects, encompassing both shortening and enlargement, may contribute to the sensory dysfunction observed in the neurodegenerative models.

### 3.4 Functional impairments of olfactory neurons of *C. elegans* models of neurodegeneration

**Figure 5:**
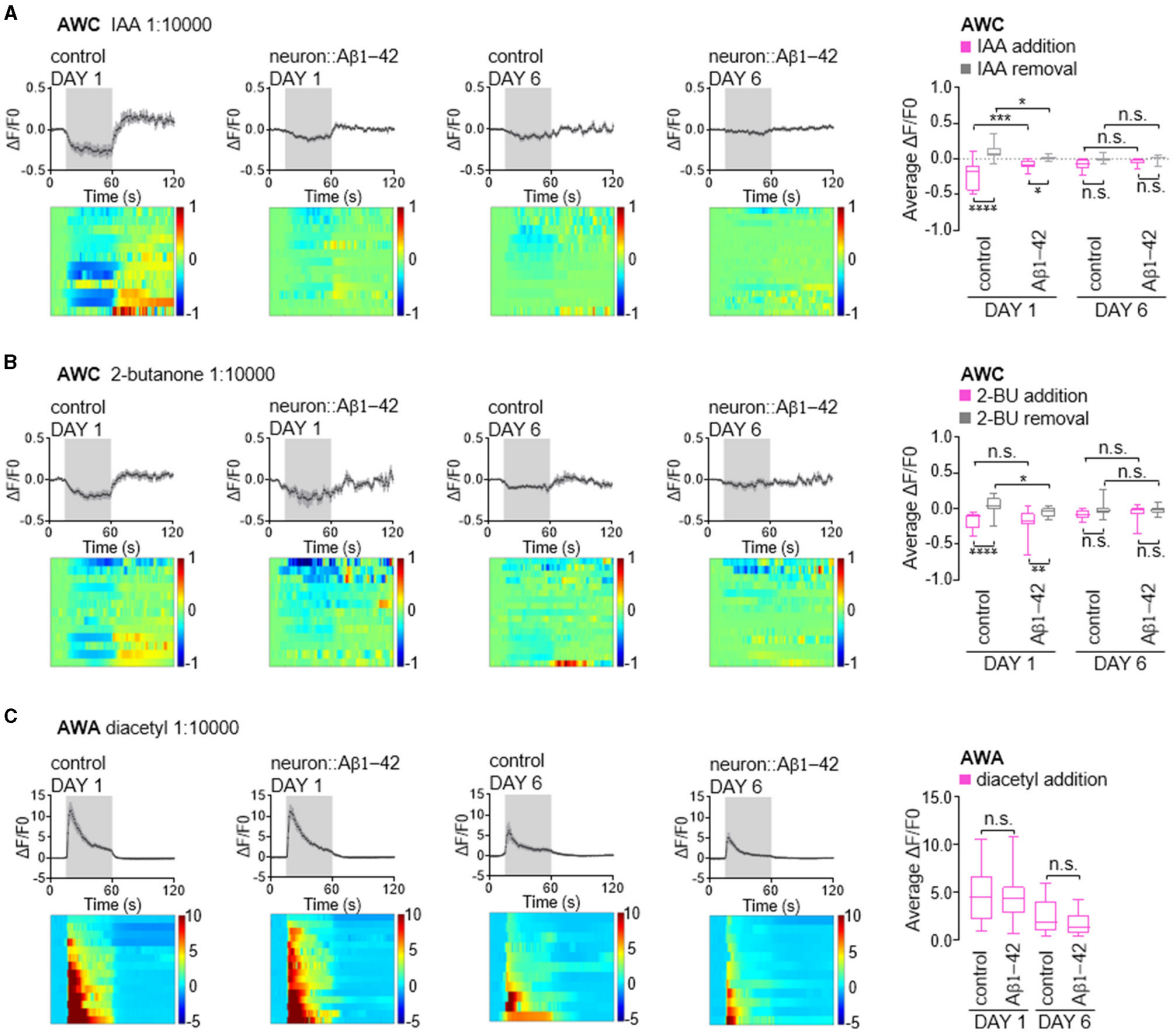
Calcium responses in AWC and AWA olfactory neurons in neurodegenerative *C. elegans* models. **(A**–**C)** Average changes and corresponding heatmaps of GCaMP fluorescence in wild-type and neurodegenerative AWC or AWA neurons of in day 1 and day 6 adult *C. elegans*. The calcium responses are elicited by odor stimulation with IAA **(A)**, 2-butanone **(B)** or diacetyl **(C)**. Quantitative data are shown on the right, presented as the 25th, 50th, and 75th percentiles, as well as the minimum and maximum values. *P* values are derived from two-way ANOVA with Sidak's multiple comparisons test. *P < 0.05; **P < 0.01; ***P < 0.001; ****P < 0.0001; n.s., not significant.

Next, we investigated whether the functional consequences of these findings are attributed to impairments in calcium responses in the olfactory neurons. AWC neurons are OFF neurons. Exposure to attractive odors typically elicits a decrease in intracellular calcium concentration, followed by a rise in calcium upon odor removal (Chalasani et al., 2007, 2010). Consistent with established response patterns, control day 1 adult animals exhibited robust calcium transients in AWC neurons upon exposure to attractive odors of IAA or 2-butanone (Figures 5A, B). In contrast, worms expressing Aβ1-42 displayed disruptions in these calcium dynamics. Notably, the decrease in calcium upon odor presentation and the subsequent increase upon odor removal were largely abolished in these animals. Interestingly, in day 6 adult animals, both control and Aβ1-42 worms exhibited diminished calcium responses compared to their day 1 counterparts (Figures 5A, B). This age-dependent decline in calcium signaling suggests a general decrease in AWC olfactory neuronal function with age, independent of Aβ1-42 expression.

AWA is an ON neuron known to exhibit elevated calcium levels in response to stimuli (Larsch et al., 2013, 2015). Unlike AWC neurons, AWA neurons showed minimal alteration in their response to diacetyl in worms expressing of Aβ1-42. These neurons typically exhibit a robust calcium increase upon exposure to diacetyl, which tapers rapidly (Figure 5C). By day 6, worms exhibited reduced calcium increases in AWA neurons in response to diacetyl; however, this response was comparable in age-matched animals expressing Aβ1-42 (Figure 5C), consistent with the absence of olfactory behavior defects (Figure 1). Given that calcium dynamics in olfactory neurons are the initial stage of olfactory stimuli signaling, our findings suggest that reduced olfactory sensitivity may be attributed to the calcium dynamics in olfactory neurons, in addition to possible synaptic defects caused by Aβ1-42 expression in *C. elegans*.

### 3.5 ER^*UPR*^ is prominently activated in in *C. elegans* with pan-neuronal expression of Aβ1-42

**Figure 6:**
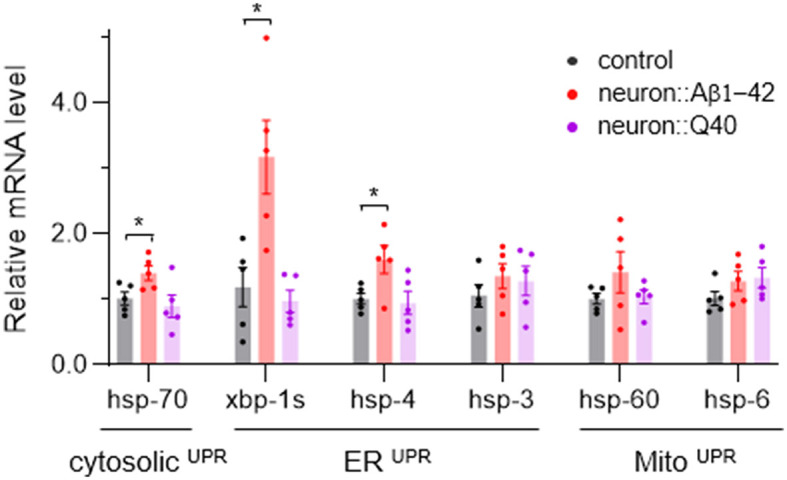
The average expression levels of several UPR-related genes in neurodegenerative *C. elegans* models. *N* = 5, Data are presented as mean ± s.e.m. *P* values are derived from Student's *t*-test. *P < 0.05.

Disruption of cellular proteostasis leads to unfolded protein responses (UPR), which can occur in different cellular compartment—cytosol, ER and mitochondria—each triggering different signaling pathways (Taylor et al., 2014; Frakes and Dillin, 2017). Given that neurodegenerative diseases are characterized by the abnormal accumulation of misfolded proteins, we investigated which UPR processes—Mito^UPR^, ER^UPR^ and cytosolic UPR—are activated in C. elegans models of neurodegeneration. We used quantitative PCR to measure the expression levels of reporter genes associated with each UPR type. Our results indicate that genes linked to all three UPR pathways were not significantly upregulated by Q40 expression, consistent with that Q40 does not form insoluble aggregates. However, ER^UPR^ was notably activated in worm expressing Aβ1-42, with spliced *xbp-1* increased more than three folds and *hsp-4* significant upregulated (Figure 6). In contrast, the cytosolic ^UPR^ marker *hsp-70* showed a slight but significant increase, while Mito^UPR^ markers *hsp-6* and *hsp-60* did not did not exhibit significant changes (Figure 6).

### 3.6 AMPK activation relieves defects in olfaction and Aβ aggregation in *C. elegans* models of neurodegeneration

**Figure 7:**
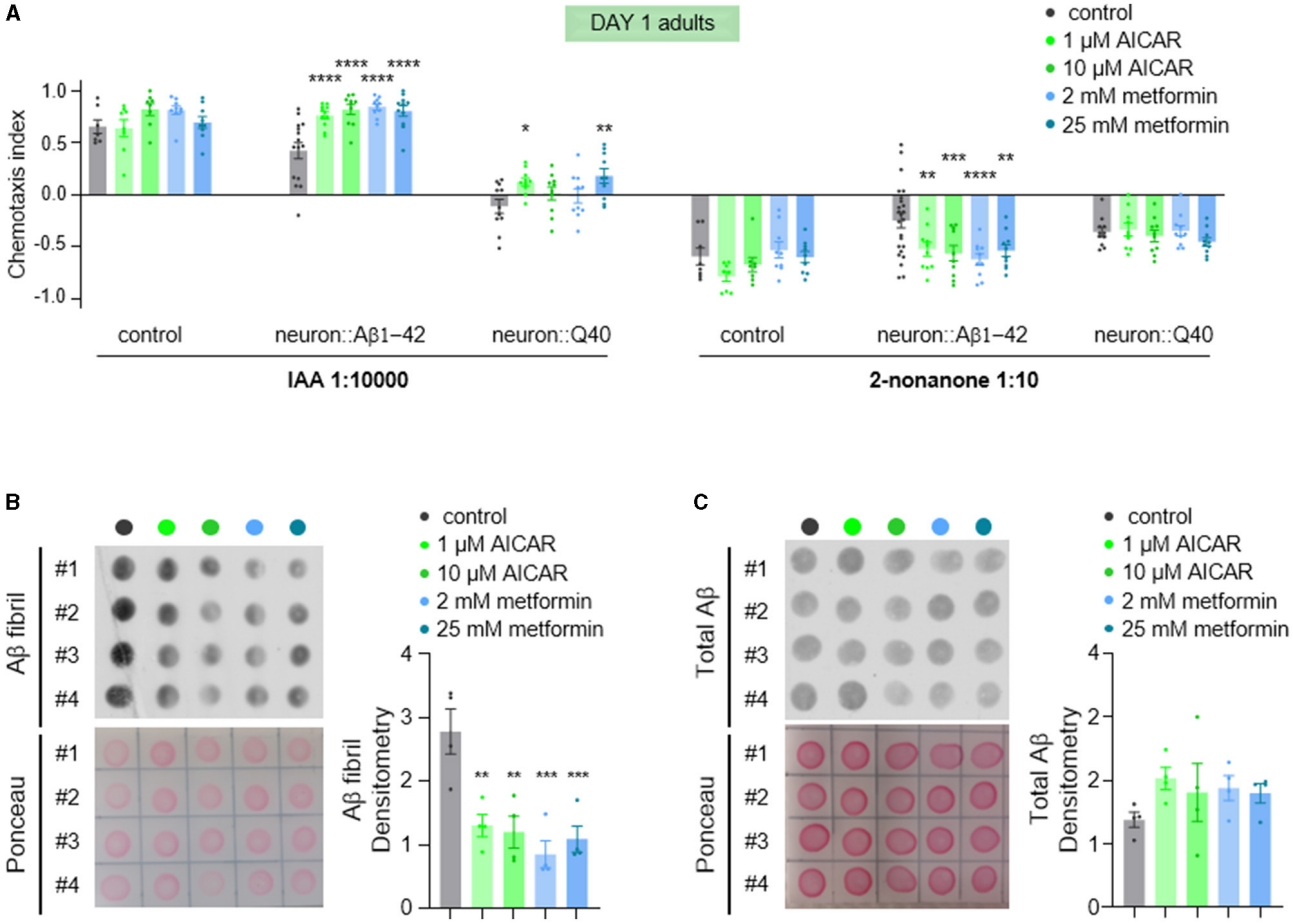
AICAR and metformin treatments alleviate olfaction defects and reduce Aβ aggregation. **(A)** Chemotaxis responses of wild-type adults and adults with pan-neuronal expression of Aβ1-42 or Q40 to IAA and 2-nonanone, following treatment with AICAR or metformin. Data from three or more independent experiments are presented as mean ± s.e.m. *P* values are derived from two-way ANOVA with Dunnett's multiple comparisons test. *P < 0.05; **P < 0.01; ***P < 0.001; ****P < 0.0001; no asterisk indicates not significant. **(B**, **C)** Fibril Aβ levels **(B)** and total Aβ levels **(C)** in worms treated with of AICAR or metformin were measured using a dot blot assay with. Total Aβ levels remained unchanged with treatments, but fibril Aβ levels decreased significantly. Aβ densitometry was quantified on the right. *N* = 4, Data are presented as mean ± s.e.m. *P* values are derived from one-way ANOVA with Dunnett's multiple comparisons test. **P < 0.01; ***P < 0.001.

AMPK (AMP-activated protein kinase) is a crucial intracellular energy sensor that maintains cellular homeostasis by responding to low energy levels (Steinberg and Hardie, 2023). It is activated by adenosine monophosphate (AMP), which indicates reduced intracellular energy level. It can also be activated by AMP analogs such as 5-aminoimidazole-4-carboxamide-1-b-dribofuranoside (AICAR), and low dose metformin. In neurodegenerative diseases, AMPK's function is particularly relevant due to the high energy demands of the brain. Metabolic stress caused by mitochondrial dysfunction was reported to precede a global imbalance in proteostasis in the *C. elegans* model expressing Aβ1-42 (Teo et al., 2019). This metabolic disturbance was rescued by the anti-diabetic drug metformin (Teo et al., 2019). Therefore, we tested whether metformin treatment could reverse the olfactory defects observed in young animals, given that metformin activates AMPK (Ma et al., 2022). Consistent with our expectations, we found that metformin significantly alleviated the olfactory defects (Figure 7A) and decreased fibrillar Aβ levels (Figures 7B, C). The involvement of AMPK activation by metformin was further supported by treatment with the specific AMPK activator, AICAR (Figure 7). These results suggest that improving metabolic stress via the AMPK signaling pathway is a potential therapeutic approach for Aβ-related neurodegenerative diseases.

## 4 Discussion

### 4.1 Characterization of neuronal dysfunction in *C. elegans* models of neurodegenerative diseases

*C. elegans* models expressing neurodegenerative disease proteins, particularly human Aβ1-42 and Q40::YFP, have been instrumental in understanding protein homeostasis mechanisms and identifying potential therapeutic drugs. These models often use muscle cell expression, leading to readily detectable phenotypes like paralysis and movement defects. Expressing Aβ1-42 in neurons causes mild movement issues and metabolic stress in the whole body. Q40 expression in the nervous system results in body bending deficits and neuronal protein aggregation. However, the specific characteristics of neural damage in terms of morphology and function remain underexplored. In this study, we examined two *C. elegans* models of neurodegeneration at multiple levels: protein aggregation, neuron morphology, functional calcium imaging, and olfactory behavior, hoping to provide a more complete picture of disease progression in *C. elegans* models.

Here, we show that both *C. elegans* models of neurodegeneration exhibit olfaction defects, characterized by reduced olfactory sensitivity to odors sensed by AWB and AWC neurons, but not AWA-sensed odors, even in day 6 adults. This suggests that the toxicity induced by protein aggregation in *C. elegans* is neuronal cell-type dependent, with AWA neurons being more resistant to the dysfunction caused by protein aggregation compared to AWC and AWB neurons.

As sensory neurons detecting food odors, AWA neurons can detect a wide range of odor concentrations spanning over 100,000-fold. Calcium imaging in AWA neurons reliably captures responses across this extensive range of odor concentrations. A unique aspect of AWA neurons is their capability to exhibit action potential-like firing, facilitated by voltage-gated calcium channels (Liu et al., 2018), unlike most neurons in *C. elegans* which lack voltage-gated sodium channels (Bargmann, 1998) and myelin sheaths, and are considered graded neurons. It is conceivable that AWA neurons possess distinctive signaling mechanisms that contribute to their wide-ranging responsiveness and resilience to proteostasis stress. Future studies investigating these unique properties hold promise for uncovering novel mechanisms of neuronal resilience to proteostasis stress.

At the molecular level, expressing either Aβ1-42 or Q40 in *C. elegans* neurons caused remarkable age-dependent protein aggregation and morphological changes. Aβ1-42 formed fibrils as early as day 1 adults. While Q40::YFP did not form insoluble aggregates, soluble aggregates began accumulating in neuron cell bodies and along neurites, becoming prominent by day 6 adults. Moreover, in both models, we observed significantly shortened cilia and a high percentage of cilia enlargement even in day 1 AWB neurons. These morphological alterations indicate early neuronal impairment and suggest that abnormal cilia may play a role in the observed olfactory deficits.

Importantly, we observed calcium signaling defects in AWC neurons expressing Aβ1-42. Day 1 adults exhibited reduced responses to both IAA and 2-butanone upon odor addition and removal. By day 6, the calcium signaling in these neurons was significantly diminished, resulting in no noticeable difference between the model worms and the controls upon odor stimulation. This finding is intriguing because human AD patients often exhibit heightened calcium signals in olfaction-related brain regions during olfaction discrimination tasks (Mormino et al., 2012; Murphy, 2019). Such heightened calcium signaling is thought to cause neuronal stress and contribute to further protein aggregation. However, in the *C. elegans* model, we observed a decrease in calcium levels occurring as early as the preliminary stages of protein aggregation. This suggests that decreased calcium signaling and reduced olfactory sensitivity are early indicators of neurodegenerative progression in the *C. elegans* AD model.

Taken together, our study underscores the importance of a multifaceted approach combining molecular, morphological, and functional analyses in neurodegenerative models to better understand the neuronal cell-type-specific effects of protein aggregation and the underlying mechanisms. We found that both calcium dynamics and olfactory behavior exhibited defects in day 1 adults expressing Aβ1-42 or Q40, even though visible protein aggregation within neurons was not yet apparent at this early stage. Our data suggest that in *C. elegans* models of neurodegeneration, olfactory dysfunction, a potential early contributor to AD and polyglutamine diseases pathology, may originate from diminished neuronal calcium signaling. This reduction sensory signal transduction, as evident by the observed enlargement of cilia morphology, could precede the formation of visible protein aggregates.

### 4.2 Insights into neurodegenerative disease pathways from *C. elegans* models

Since protein aggregation often causes unfolded protein responses (UPR), we assayed the occurrence of Mito^UPR^, ER^UPR^ and cytosolic ^UPR^ by analyzing common reporters. Worms expressing Q40 did not show significant upregulation of any UPR markers. However, worms expressing Aβ1-42 exhibited strong activated ER^UPR^, evidenced by significant upregulation of spliced *xbp-1* and *hsp-4*. There is a slight but significant increase of *hsp-70*, which indicates activation of cytosolic ^UPR^. We cannot rule out Mito^UPR^ activation as our assay was performed on whole bodies, potentially diluting neuronal-specific changes with data from unaffected tissues.

AMPK activation is known to induce autophagy, aiding in the clearance of protein aggregates (Agostini et al., 2023). As a key cellular energy sensor, AMPK also plays a critical role in maintaining homeostasis in the nervous system (Bobela et al., 2017; Muraleedharan and Dasgupta, 2022; Li et al., 2024). In this study, treatment with AMPK agonists AICAR and metformin significantly relieved olfactory defects and reduced Aβ aggregation in neurodegenerative *C. elegans* models. Their impact on Q40-induced olfactory defects was modest, possibly due to the severe nature of the defects in these worms. Overall, our findings suggest that AICAR and metformin may offer therapeutic potential for neurodegenerative diseases by activating AMPK.

### 4.3 Pros and cons of *C. elegans* models in studying neurodegenerative diseases

Studying neurodegenerative diseases like AD and HD using complex mouse models is expensive and time-consuming. *C. elegans*, a simple worm with a rapid life cycle and easily manipulated genes, offers a powerful alternative. Its well-defined nervous system and genetic tractability make it a cost-effective platform for such research. Genetic and drug screenings in *C. elegans* Aβ models havev identified potential treatments. For instance, a targeted RNAi screen using a transgenic *C. elegans* strain expressing secretory Aβ1-42 identified collagens as modifiers to enhance or to attenuate Aβ aggregation and ADM-2, a metalloprotease, as a key extracellular factor to remove Aβ (Jongsma et al., 2023). Natural products like *Holothuria scabra* and *Radix Stellariae* extracts, along with D-Pinitol, reduce Aβ aggregation and ROS levels (Azab, 2022; Kleawyothatis et al., 2022; Long et al., 2023). *Ginkgo biloba* extract EGb 761 has shown promise in mitigating pathological features in *C. elegans* models expressing Aβ1-42 (Wu et al., 2006). Similarly, a genetic screen identified *polyQenhancer-1* (*pqe-1*) as a protective factor against HD neurotoxicity. RNAi screens have uncovered 88 genetic suppressors of polyQ aggregation and toxicity (Silva et al., 2011) and 49 modifiers of 128Q-mediated neuronal dysfunction, findings that align with observations in HD mice models (Lejeune et al., 2012). Using ethyl methanesulfonate (EMS) to induce mutations in *C. elegans*, van Ham and colleagues identified MOAG-4 as a positive regulator of polyQ aggregation formation (Ham et al., 2010), showing that inactivation of MOAG-4 significantly reduced aggregates. Importantly, MOAG-4 is evolutionarily conserved, and its human orthologs, SERF1A and SERF2, can enhance polyQ aggregation.

*C. elegans* serves as a powerful tool for exploring neurodegenerative diseases and conducting high-throughput drug screening. However, its simplicity presents limitations. The worm's nervous system lacks the complex architecture of mammals, such as structures like the caudate and putamen, which are important for understanding polyglutamine diseases such as HD. Additionally, *C. elegans* lacks myelin sheaths and an adaptive immune system, which are crucial for replicating aspects of human neurodegenerative disease pathology, including neuroinflammation, a significant feature in AD pathogenesis. Despite these limitations, drug candidates identified in *C. elegans* models show promise and are actively being evaluated for efficacy and relevance in mammalian systems. This highlights the importance and usefulness of *C. elegans* models for neurodegenerative diseases in advancing our understanding of the molecular, cellular, and genetic mechanisms underlying neurodegenerative diseases and guiding the development of new therapeutic strategies.

## Data Availability

The original contributions presented in the study are included in the article/Supplementary material, further inquiries can be directed to the corresponding authors.
